# Using the Judgment Bias Task to Identify Behavioral Indicators of Affective State: Do Eye Wrinkles in Horses Reflect Mood?

**DOI:** 10.3389/fvets.2021.676888

**Published:** 2021-07-08

**Authors:** Sara Hintze, Lisa Schanz

**Affiliations:** Division of Livestock Sciences, Department of Sustainable Agricultural Systems, University of Natural Resources and Life Sciences, Vienna, Austria

**Keywords:** animal welfare, animal emotion, facial expression, eye wrinkle expression, cognitive bias task

## Abstract

Identifying and validating behavioral indicators of mood are important for the assessment of animal welfare. Here, we investigated whether horses' eye wrinkle expression in a presumably neutral situation is a measure of mood as assessed in a cognitive judgment bias task (JBT). To this end, we scored pictures of the left and right eyes of 16 stallions for different aspects of eye wrinkle expression and tested the same individuals on a spatial JBT with active trial initiation. Eye wrinkle expressions were assessed by a qualitative assessment, i.e., the overall assessment of how “worried” horses look, the number of wrinkles, and the angle measured at the intersection of lines drawn through the eyeball and the topmost wrinkle. Correlations between the three eye wrinkle measures and the optimism index as a measure of horses' decisions in the JBT were not statistically significant, but with increasing optimism index, horses tended to be scored as looking less worried (qualitative assessment). We discuss our findings from different perspectives and make suggestions for future research, e.g., by calling for experimental induction of mood and thus greater variation within and/or between individuals and by investigating the interplay between shorter-lasting emotional and longer-lasting mood states to further explore the potential use of the JBT to validate eye wrinkles and other facial or body expressions as indicators of mood.

## Introduction

Measures of spontaneous behavioral expression, including facial expressions, have great potential for assessing animals' affective states since they are non-invasive and do not require animals to be trained. This research area is still in its infancy (1), but has received increasing attention in recent years (2). One approach to study facial expressions in non-human animals is the Facial Action Coding System (FACS), which details all potential expressions resulting from facial muscle contraction and relaxation. Originally developed for humans (3), FACSs have been adapted to different species encompassing different primates [e.g., (4, 5)], horses (6), dogs (7, 8), and cats (9, 10). They serve as a tool to study facial expressions across different contexts. Another approach is to identify context-specific changes in the face by comparing, for example, facial expressions in a painful and a control situation. So-called pain grimace scales have been developed and applied in a number of species and across various painful situations [e.g., (11–14)]. Facial expressions have also been studied as potential indicators of other negative and positive affective states. Most studies compared changes in the face during or immediately after exposure to a presumably positive or negative stimulus to a presumably neutral control situation, focusing on either several parts of the face [e.g., (15, 16)] or only aspects, for example, the ears [e.g., (17, 18)] or eyes [e.g., (19, 20)]. The identified facial expressions are supposed to reflect stimulus-specific short-term affective states, i.e., emotions. Research on facial expressions indicative of longer-term affective states, i.e., moods, is less readily available, mainly because of the quick transitions between contraction and relaxation of facial muscles. However, some studies show that facial expressions are also indicative of chronic pain [e.g., dental disorders in horses: (21); footrot in sheep: (22)]. Beyond the context of pain, researchers discuss whether facial expression may indicate not only short-term discrete emotional states but also possibly emotional valence (23) and long-term mood (24). Research in humans revealed that people suffering from psychological disorders like borderline personality disorders (25) or (unipolar) depression (26, 27) show reduced facial expressiveness (25, 26) or overall different patterns of facial expression (27) when exposed to emotional movies or memories, indicating an interaction between underlying long-term affective states and facial expression of emotion.

Facial expressions reflecting long-term affective states would be highly valuable as indicators for the assessment of animal welfare. Here, we present a first study investigating one particular aspect of facial expression, eye wrinkle expression in horses, as a potential indicator of mood. Eye wrinkles above the eyeball originate from the contraction of the inner brow raiser and have been described in detail in two studies. In the first study, eye wrinkles were assessed in horses exposed to two presumably positive and two presumably negative situations (28). The angle, one measure of eye wrinkle expression, increased in one negative situation (caused by muscle contraction) and decreased in one positive situation (caused by muscle relaxation), while other measures remained unaffected. In the second study, the effect of age, sex, breed type, and coat color on eye wrinkle expression in a presumably neutral situation was assessed in 181 horses (29). Except for breed type affecting the width of the angle, none of the investigated factors explained the high variation in eye wrinkle expression between individuals; differences in mood could be a potential explanation (29). Eye wrinkles develop or deepen when the “skin above the inner corner of the eye is pulled dorsally and obliquely toward the medial frontal region,” which is defined as Action Unit 101 in the equine FACS [(6), p. 8], similar to Action Unit 1 in the human FACS (3). In humans, Action Unit 1 is activated when a person is sad [e.g., (24, 30)], and it forms part of the “Veraguth fold,” a fold above the eyes which appears when the inner eyebrows are lifted and pulled together in depressed individuals [e.g., (31, 32)], suggesting a relationship between a contraction of the inner brow raiser and negative mood, at least in humans.

The present study aimed to investigate the relationship between eye wrinkle expression and mood in horses. To this end, we assessed eye wrinkles in 16 stallions on pictures taken in their home environments and tested the same individuals on a judgment bias task (JBT) as a measure of mood. In a JBT, decision making under ambiguity is examined, and animals' responses to the ambiguous cues are interpreted as either “optimistic” (i.e., the animal responds as if expecting a positive outcome) or “pessimistic” (i.e., the animal responds as if expecting a negative outcome). For details on the background and theory of the JBT, see the (systematic) reviews by Mendl et al. (33), Roelofs et al. (34), Neville et al. (35), and Lagisz et al. (36). If horses' eye wrinkles are an indicator of mood, we predicted that with decreasing scores in eye wrinkle expression, horses will be more likely to respond optimistically by showing go responses when exposed to ambiguous trials in the JBT.

## Animals, Materials, and Methods

### Animals

This study includes data from 16 Franches-Montagnes stallions between 4 and 22 years of age (mean ± standard deviation: 9.1 ± 4.8 years). Horses were housed in standard single boxes (3 × 3.5 m) on wood shavings or straw with visual contact to conspecifics at the Swiss National Stud Farm of Agroscope (Avenches, Switzerland). For more information, see Hintze et al. (37).

### JBT

The detailed training and test protocols as well as the individual performance of the horses during testing are described in Hintze et al. (37). In short, we used a spatial task design with the location of two out of five goal-boxes signaling either reward or non-reward. The opening of one of the goal-boxes served as a cue for the horses. Before the discrimination training, horses learned to actively initiate each trial by touching a bottle opposite the goal-boxes. After reaching the learning criterion (i.e., 80% correct responses in positive and negative trials across two consecutive sessions), horses were exposed to six test sessions, in each of which they were confronted with three intermediate cues (near positive, middle, and near negative) interspersed between 25 positive and 25 negative reference cues.

### Eye Wrinkle Assessment

Pictures from the eye area of the horses were taken in their home boxes approximately 2 weeks after JBT testing had finished. The experimenter who had trained and tested the horses and was thus familiar to them (SH) took all pictures in a presumably neutral situation, i.e., between noon and 2 p.m. when it was most quiet at the stud farm. Moreover, she stopped the process if any visual or acoustic disturbance occurred. All details of the picture-taking process are described in Schanz et al. (29). We randomly chose six pictures per horse, three from the left eye area and three from the right eye area. All 96 pictures were scored according to the eye wrinkle assessment scale by Schanz et al. (29). This scale includes five outcome measures: (1) qualitative assessment, “qualitative”, i.e., overall first assessment of how “worried” the horse looks assessed on a visual analog scale ranging from 0 to 100 (“not worried” to “extremely worried”); (2) “brow raised”, i.e., the amount the skin above the eye is raised assessed on a visual analog scale ranging from 0 to 100 (“not raised” to “strongly raised”); (3) “number”, i.e., the number of wrinkles above the eyeball; (4) “markedness”, i.e., depth and width of the wrinkles assessed in the three categories “no wrinkles”, “weak”, “strong”; and (5) “angle” measured in degree at the intersection of lines drawn through the eyeball and the topmost wrinkle.

### Ethical Considerations

This study was carried out in accordance with the guidelines of the Swiss Animal Welfare Ordinance (TSchV 455.1). The experimental procedures were approved by the Cantonal Veterinary Office in Vaud, Switzerland (license number: 2804.1).

### Statistical Analyses

Analyses were performed using the statistical programming language R [R version 4.0.2, (38); RStudio version 1.4.1103, (39)]. The graph was produced using the package “ggplot2” (40).

#### Collinearity Between Eye Wrinkle Measures

The eye wrinkle data we used are a subset of the data published by Schanz et al. (29), in which collinearity between some of the measures was found. We performed the same checks for the subset, i.e., Spearman's rank correlations to assess the association between two continuous measures and Kruskal–Wallis tests with subsequent pairwise Wilcoxon rank sum tests to assess the relationship between one continuous and one categorical measure. A strong correlation between “qualitative” and “brow raised” (*r*_s_ = 0.92, *p* < 0.001) was found; “brow raised” was excluded from further analyses. “Qualitative” ($\chi_{2}^{2}$ = 31.43, *p* < 0.001), “brow raised” ($\chi_{2}^{2}$ = 33.22, *p* < 0.001) and “number” ($\chi_{2}^{2}$ = 86.07, *p* < 0.001) were associated with “markedness,” and thus, “markedness” was excluded from further analyses.

#### Differences in Eye Wrinkle Measures for the Left and the Right Eyes

The mean score per eye and horse was calculated for each eye wrinkle measure to test for a possible difference between the left and the right eyes. Linear mixed-effects models [function: lme, package: nlme, (41)] were run with “eye” (left or right) as a fixed effect and “horse” as a random effect. Model assumptions were verified by visually checking residuals for normal distribution and homogeneity of variance. Transformation of the data was not necessary.

#### Correlations Between Eye Wrinkle Measures and Performance in the JBT

To correlate the results from the JBT with the eye wrinkle measures, we first calculated an “optimism index” for each horse [see (42)]. In short, for ambiguous trials, the proportion of no-go responses was subtracted from the proportion of go responses, resulting in one value ranging between −1 and 1 per horse. Normal distribution of the “optimism index” and the eye wrinkle measures was visually checked; only the “optimism index” was normally distributed. We thus ran Spearman's rank correlations for the three eye wrinkle measures and the “optimism index”. For “angle”, a subset of the full data set was used; only pictures with at least one wrinkle (“number” ≥ 1) were included since an angle could only be measured if at least one wrinkle was identified (28, 29).

## Results

### Overview of the Proportion of Go Responses in the JBT

The proportion of go responses ranged from 0.5 to 1 in near-positive trials (mean ± standard deviation: 0.84 ± 0.2), from 0.3 to 1 in middle trials (0.80 ± 0.2) and from 0 to 1 in near-negative trials (0.36 ± 0.3). The “optimism index” across the three ambiguous cues ranged from −0.43 to 1 (0.31 ± 0.41). Information on training duration and test performance can be found in Hintze et al. (37).

### Overview of the Eye Wrinkle Measures

“Qualitative” ranged between 0.5 and 100 on a visual analog scale ranging from 0 to 100 (mean ± standard deviation: 45.8 ± 30.9), “number” of wrinkles varied between 0 and 4 (0.8 ± 1.0), and the “angle” ranged from 18.1 to 38.8° (30.9 ± 7.2).

### No Differences in Eye Wrinkle Measures Between the Left and the Right Eyes

No statistically significant difference between eyes was found for “qualitative” (*F*_1, 15_ = 1.51, *p* = 0.24), “number” (*F*_1, 15_ = 1.71, *p* = 0.21), or “angle” (*F*_1, 7_ = 0.61, *p* = 0.46).

### Correlations Between Eye Wrinkle Measures and Performance in the JBT

**Figure 1:**
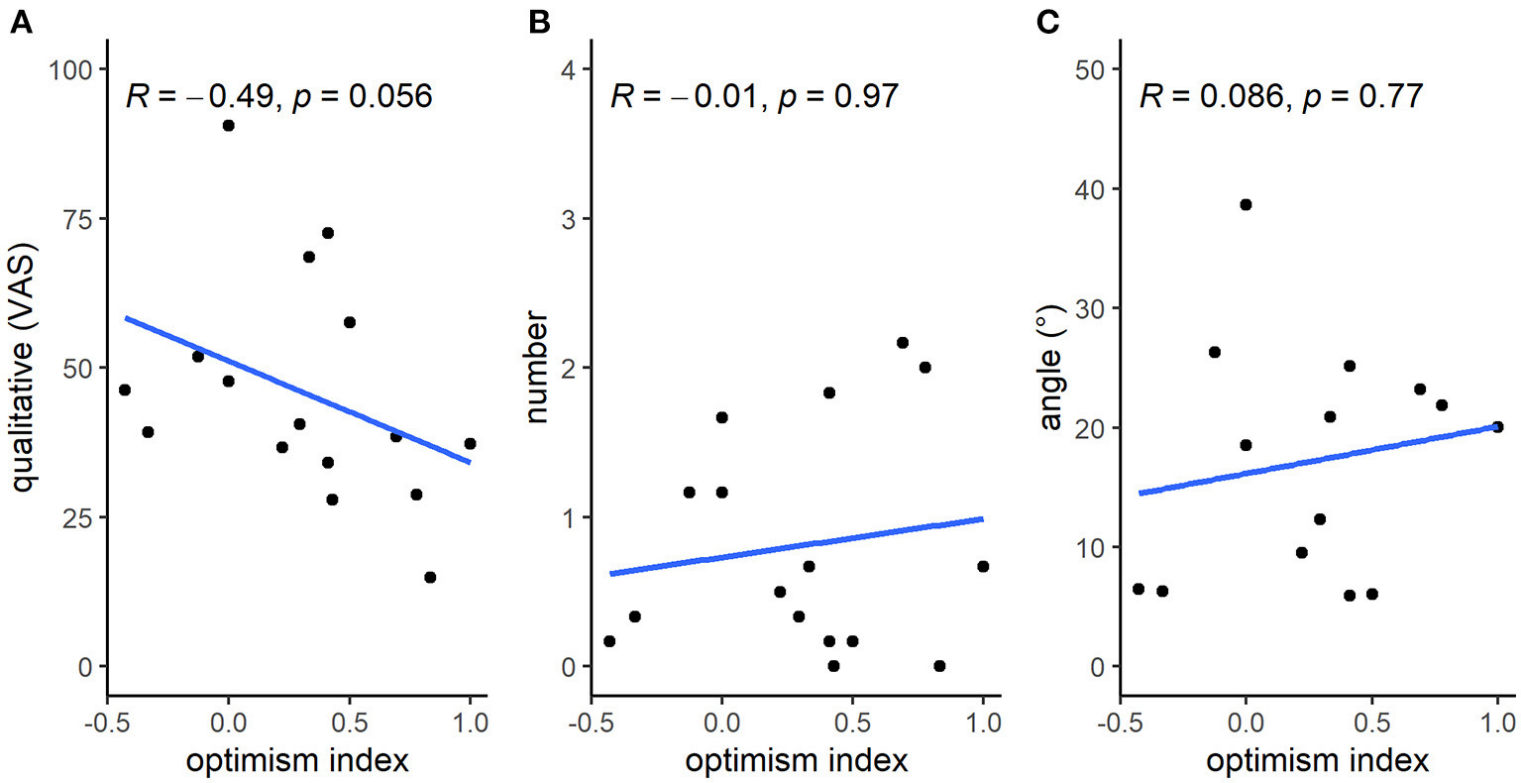
Correlations between each eye wrinkle measure and the “optimism index”. Spearman's rank correlation coefficients rho (*R*) with their associated *p*-values and visualizations (blue line) based on the displayed data points are given for the eye wrinkle measures “qualitative” **(A)**, “number” **(B)**, and “angle” **(C)**.

The correlations between the three eye wrinkle measures and the “optimism index” were not statistically significant (“qualitative”: *r*_s_ = −0.49, *p* = 0.06; “number”: *r*_s_ = −0.01, *p* = 0.97; “angle”: *r*_s_ = 0.09, *p* = 0.77; Figure 1), but there was a tendency for a moderate negative correlation between “qualitative” and the “optimism index”; i.e., with an increasing “optimism index”, horses tended to be scored as looking less worried.

## Discussion

We aimed to investigate whether horses' eye wrinkle expression is a potential indicator of mood as assessed in a spatial JBT with active trial initiation. Correlations between the three eye wrinkle measures and the “optimism index” as a measure of horses' performance in the JBT were not statistically significant, but with an increasing “optimism index”, horses tended to be scored as looking less worried (“qualitative”).

The statistical tendency for the moderate negative correlation between the qualitative assessment and horses' decisions in the JBT is in line with our prediction that horses respond less optimistically in the JBT with higher scores for the eye wrinkle expression. This tentatively indicates that one aspect of eye wrinkle expression may reflect mood in horses. However, this result needs to be interpreted carefully since our sample size was relatively small, which increases the risk of obtaining a spuriously large correlation coefficient.

Our statistically non-significant results need to be discussed from different perspectives. First, it is possible that some aspects of eye wrinkle expression, like the angle, are affected by short-term emotional states, as shown by Hintze et al. (28), rather than by longer-term mood states. However, we know that eye wrinkles are also visible in presumably neutral situations (29) and control situation (28) and not only in response to short-term stimuli. Moreover, contraction of the inner brow raiser may potentially be caused by general eye movement. A recent study in dogs demonstrated that “puppy dog eyes”, also resulting from contraction of the inner brow raiser, could be caused by movement of the eyes and that the expression was shown independently of eye movement only in 6% of the cases (43). It would thus be valuable to investigate the relationship between eye wrinkle expression and eye movement in horses as well. However, the puppy-like expression in dogs was only compared across different emotional situations and not to a control situation (43, 44); thus, we do not know whether the expression is also present in rather neutral situations as in the case of horses. Eye wrinkle expression in neutral situations may potentially be less affected by eye movement than during emotional situations, but further research is needed to investigate this association.

Second, the variation between horses' performance in the JBT and in the eye wrinkle measures might have been too small to detect potential associations. All measures were taken in presumably neutral situations without the horses' mood being manipulated by a treatment. A next step would thus be to include a mood-inducing treatment, e.g., varying housing conditions, potentially resulting in greater variation between and, depending on the experimental design, within animals. Moreover, it would be worth considering how underlying mood states affect changes in facial expressions when confronted with short-term stimuli and thus the interaction between longer-term mood and shorter-term emotional states. This suggestion is in line with the results from a recent study by Clarkson et al. (45), who investigated the interplay between mood and emotions and found that negative mood affected the expression of negative but not positive emotion in mice. It would therefore be valuable to assess changes in response to positive and negative short-term stimuli in addition to the implementation of a mood-inducing treatment.

Third, in a correlational approach, it is always important to discuss both variables of interest. Mood may not have affected eye wrinkle expression, but it is also possible that the JBT was not sensitive enough to detect only slight differences in mood states between the horses. So far, to our knowledge, the JBT has been used primarily to detect differences between mood-inducing treatments but not to draw inferences from the animals' baseline mood. Moreover, we calculated an “optimism index” for correlation with the eye wrinkle measures, which accounts for the total number of go and no-go responses in ambiguous trials but ignores the different trial types (i.e., near positive, middle, and near negative); thus, it may not be sensitive enough to detect subtle differences in mood.

Fourth, the temporal delay between the JBT testing and the picture-taking process was not ideal and could potentially explain why we did not find an association between the measures since the stallions' mood may have changed over this time. However, treatment and management of the horses did not change during these two weeks, which is why we do not have an indication that the horses' mood changed, but the methodological limitation of the temporal delay needs to be acknowledged.

## Conclusion and Outlook

Previous research has shown that eye wrinkle expression is independent of most characteristics of the horse and that the angle measured at the intersection of lines drawn through the eyeball and the topmost wrinkle may be an indicator of short-term emotional states. The present study does not support our hypothesis that eye wrinkle expression in horses may serve as an indicator of mood as assessed in a JBT, but the tendency for a statistically significant, moderate, and negative correlation between the qualitative assessment of the eye wrinkles and the “optimism index” as a measure of judgment bias warrants further investigation. Future studies using a mood-inducing treatment and investigating the interplay between shorter-lasting emotional and longer-lasting mood states are needed to explore the potential use of the JBT to validate eye wrinkles and other facial as well as body expressions as indicators of mood.

## Data Availability Statement

The original contributions presented in the study are included in the article/Supplementary Material, further inquiries can be directed to the corresponding author/s.

## Ethics Statement

The animal study was reviewed and approved by the Cantonal Veterinary Office in Vaud, Switzerland (license number: 2804.1).

## Author Contributions

SH trained and tested the horses on the judgement bias task, took the pictures from the horses' eye, and wrote the first draft of the manuscript. LS scored all pictures, performed the data analysis, and edited the manuscript. All authors contributed to the article and approved the submitted version.

## Conflict of Interest

The authors declare that the research was conducted in the absence of any commercial or financial relationships that could be construed as a potential conflict of interest. The reviewer MS declared a past co-authorship with one of the authors SH to the handling editor.
